# Chronic toxicity of tire and road wear particles to water- and sediment-dwelling organisms

**DOI:** 10.1007/s10646-012-0998-9

**Published:** 2012-09-22

**Authors:** Julie M. Panko, Marisa L. Kreider, Britt L. McAtee, Christopher Marwood

**Affiliations:** 1 ChemRisk, 20 Stanwix Street, Suite 505, Pittsburgh, PA 15222 USA; 2 NovaTox Inc., 10 Crane Avenue, Guelph, ON N1G 2R2 Canada

**Keywords:** Tire, Particle, Chironomus, Hyalella, Daphnia, Pimephales, Sediment, Toxicity

## Abstract

Tire and road wear particles (TRWP) consist of a complex mixture of rubber, and pavement released from tires during use on road surfaces. Subsequent transport of the TRWP into freshwater sediments has raised some concern about the potential adverse effects on aquatic organisms. Previous studies have shown some potential for toxicity for tread particles, however, toxicity studies of TRWP collected from a road simulator system revealed no acute toxicity to green algae, daphnids, or fathead minnows at concentrations up to 10,000 mg/kg under conditions representative of receiving water bodies. In this study, the chronic toxicity of TRWP was evaluated in four aquatic species. Test animals were exposed to whole sediment spiked with TRWP at concentrations up to 10,000 mg/kg sediment or elutriates from spiked sediment. Exposure to TRWP spiked sediment caused mild growth inhibition in *Chironomus dilutus* but had no adverse effect on growth or reproduction in *Hyalella azteca*. Exposure to TRWP elutriates resulted in slightly diminished survival in larval *Pimephales promelas* but had no adverse effect on growth or reproduction in *Ceriodaphnia dubia.* No other endpoints in these species were affected. These results, together with previous studies demonstrating no acute toxicity of TRWP, indicate that under typical exposure conditions TRWP in sediments pose a low risk of toxicity to aquatic organisms.

## Introduction

The potential effects of road dust and its constituents on aquatic species has been investigated by various researchers and tires have been implicated as a potential toxicant (Gualtieri et al. 2005b; Mantecca et al. 2007; McKenzie et al. 2009; Stephensen et al. 2005; Wik 2007; Wik and Dave 2005, 2006, 2009; Wik et al. 2009). To date, most toxicological studies on tires have focused on tread particles (TP) generated from shredded/powdered tire tread or extracts of tire tread (Gualtieri et al. 2005a, b; Henkelmann et al. 2001; Wik 2007; Wik and Dave 2005, 2006; Wik et al. 2009). The toxicity of TP in aquatic species has been attributed to both inorganic and organic constituents of tires, although TP may vary in toxicity due to tire formula and the methods used to prepare and extract the particles (Gualtieri et al. 2005a, b; Nelson et al. 1994; Wik 2007; Wik and Dave 2006; Marwood et al. 2011).

During use, tires wear and form tire and road wear particles (TRWP) that are dispersed into the environment. As a result of the interaction of tires with road surface, TRWP consist of a complex mixture of rubber, with both embedded asphalt and minerals from the pavement, as well as free pavement. TRWP are both morphologically and chemically distinct from TP, and therefore may have a different toxicological profile than TP (Kreider et al. 2010). Marwood et al. (2011) characterized the acute aquatic toxicity of TRWP collected in isolation from other components of road dust and determined that at doses up to 10,000 mg/kg sediment in sediment, TRWP are not acutely toxic to aquatic species (*Pseudokirchneriella subcapitata*, *Daphnia magna*, and *Pimephales promelas*) under environmentally relevant conditions (Marwood et al. 2011). Only under circumstances in which the TRWP/sediment mixture was heated to high temperatures did toxicity occur in any species.

The process of heating rubber promotes the release of chemicals from the rubber matrix; these chemicals may have an effect on aquatic species that might not otherwise occur under typical environmental conditions. In fact, the results of Marwood et al. (2011) under heated conditions are consistent with previous aquatic toxicity studies where tread particles were subjected to harsh conditions (e.g. heating, organic extraction, acidic pH, etc.) to promote release of rubber chemicals (Wik and Dave 2006; Mantecca et al. 2007; Gualtieri et al. 2005a, b). Wik and Dave (2006) evaluated extracts of 25 different tire formulations (extracted at 44 °C for 72 h) and reported 48-h EC50s for tread particles ranging from 500 to >10,000 mg/L in *Daphnia magna* (Wik and Dave 2006). While the extraction conditions used in these studies may not be relevant to acute exposures in the environment, the results of these studies may indicate the potential for chronic toxicity to aquatic species from TRWP, either from extended exposure to low concentrations of chemicals or from leaching of chemicals over longer periods of time. To date no research has been conducted to investigate the potential for chronic toxicity of TRWP under environmentally relevant conditions.

The purpose of this study was to determine if TRWP are toxic to water or sediment-dwelling organisms following chronic exposure. To investigate this question, four freshwater aquatic organisms, including *Ceriodaphnia dubia*, *Pimephales promelas*, *Chironomus dilutus*, and *Hyalella azteca*, were exposed to either an elutriate of sediment spiked with 10,000 mg/kg TRWP or the spiked sediment itself and evaluated according to standard test guidelines for chronic aquatic toxicity. Sediment and sediment elutriate were selected as the exposure media based on the expectation that sediment represents the primary reservoir for TRWP in the environment (Wik and Dave 2009). For sediment elutriate studies, our methods were similar to Marwood et al. (2011) and consistent in concept to that described for partially soluble, multi-component substances (e.g., oils, creosote, etc.) where the water-accommodated fractions (WAF) of the substances containing the fraction that is dissolved or present as a stable dispersion/emulsion are assessed (OECD 2000). Studies on all organisms were consistent with standard guidance provided by the Organization for Economic Cooperation and Development (OECD) and U.S. Environmental Protection Agency (U.S. EPA) (OECD 1992, 2004; U.S. EPA 2000, 2002).

## Methods

### TRWP collection

TRWP were collected at a road simulator laboratory located within the Bundesanstalt für Straßenwesen (BASt), the German Federal Highway Research Institute, as previously described by Kreider et al. (2010). Briefly, the laboratory used an interior drum testing system containing actual asphalt pavement in cassettes. This system was electronically programmable to mimic a variety of driving conditions by varying speed, temperature, acceleration, braking, and steering. For the TRWP collection, the pavement consisted of a standardized asphalt concrete with 6.1 % proportion of bitumen (B50/70) according to ISO 10844. To prevent overheating from friction, the road surface temperature was maintained at approximately 20 °C for summer tires and 14 °C for the winter tire using a climate control system in the drum during the tests. The TRWP was collected using a vacuum system mounted behind one of the simulator wheels. Both summer and winter silica based tires (Michelin Pilot Primacy 225/55 R16 95W and Pirelli Sottozero 225/55 R16 95W M + S) and a carbon-black based summer tire (Bridgestone Potenza RE 88 205/65 R15 94W) were used to generate the TRWP. Particles from each tire were combined to form a single composite (2:1:1 Bridgestone: Michelin: Pirelli) that was sieved at 150 μm to remove any large pavement pieces. The TRWP was shipped on ice in amber glass jars to the toxicity testing laboratory under full chain of custody and were thereafter stored in the dark at 4 °C.

### Sediment and elutriate preparation

Freshwater reference sediment was collected from a drinking water reservoir near Dillon Beach, CA. This reservoir has previously been used by the toxicity testing laboratory as a source of reference sediment due to the isolation and absence of any known sources of contamination and no major drainage inputs. Analytical testing of the sediment indicated metals concentrations were generally below sediment screening criteria. To prepare the mixtures for use in toxicity testing, the reference sediment was spiked with 10 g/kg (10,000 ppm by weight) of TRWP and homogenized using mechanical mixing for 15 min. The sediment was allowed to equilibrate in cold storage (4 °C) for 48 h. Unspiked reference sediment was also mechanically mixed to control for any effects associated with the mixing process. Both mixed and unmixed reference sediment were used as controls in whole sediment toxicity tests. The spiked sediment was used for whole sediment toxicity tests in *Chironomus dilutus* and *Hyalella azteca*, and was also used to prepare the sediment elutriate used for treatment of *Ceriodaphnia dubia* and *Pimephales promelas*. All chronic toxicity tests were performed by Nautilus Environmental, LLC (San Diego, CA).

To test chronic toxicity in water-dwelling organisms, a sediment elutriate design was used. Elutriate designs for preparing test solutions that replicate sediment mobilization and storm water phenomena are recommended and have been successfully employed by other investigators for exposing water column species such as algae and daphnids (ASTM 2011; Bosch et al. 2009; Novelli et al. 2006). To prepare the sediment elutriate, one part sediment was mixed with four parts of moderately hard water for a period of 24 h at 15 °C using stainless steel mechanical mixers. After mixing, the sediment was allowed to settle for 24 h, at which time the elutriate was siphoned off and centrifuged at 1,100×*g* for 15 min. The supernatant was removed and used as the test and renewal material for the *Ceriodaphnia dubia* and *Pimephales promelas* exposures.

### Whole sediment exposures

#### *Chironomus dilutus*

*Chironomus* egg cases were obtained from Aquatic BioSystems (ABS) in Fort Collins, CO and acclimated to test conditions prior to test initiation. During acclimation, the test organisms were observed for any indications of stress, as indicated by deterioration of the egg cases. Water quality parameters including conductivity, temperature, dissolved oxygen, and pH were monitored daily. The egg cases were kept at test temperature and monitored daily until larvae hatched and vacated the egg case, at which time the test was initiated (<8-h post-hatch).

*Chironomus* larvae were exposed to reference sediment (both mixed and unmixed) or sediment containing 10,000 mg/kg TRWP for 35 days and evaluated for survival, growth, and emergence in accordance with the Organization for Economic Cooperation and Development (OECD) Technical Guidance Document (TGD) 218 (OECD 2004). In addition, laboratory control sediment consisting of a combination of Scripps sand and peat was used as a control. Each control and test sediment sample was evaluated in 10 replicates. An additional replicate was included for each control and test sample as a surrogate for measurement of water quality parameters, which occurred daily. The test chamber contained 2 cm of sediment and 400 mL of moderately hard water. A Zumwalt water renewal system was used to facilitate water renewals without disturbing the sediment (U.S. EPA 2000). Each chamber was supplied with a continuous aeration rate of three to four bubbles per second with a glass pipette. The test was conducted in an environmental chamber maintained at 23 ± 1 °C under a 16 h (light):8 h (dark) cycle. Each test chamber contained 12 larvae.

On day 10, sediments in five test chambers were removed and sieved through a 0.5 mm screen, and the number of larvae surviving was recorded. Surviving organisms were dried at 60 °C for 24 h and the dry weight of each replicate was measured to the nearest 0.01 mg. Samples were then placed in a furnace at 550 °C for 24 h to ash organic material and reweighed to provide an ash weight. The difference between the two measurements (dry weight minus ash weight) represents the organic mass of the larvae (ash-free dry weight [AFDW]).

The remaining five replicates were used to evaluate *Chironomus* emergence. Beginning on day 20 and until the end of the test period (day 35), the number and sex of emerged adult *Chironomus* were recorded daily. The test series was terminated after five consecutive days of non-emergence from the laboratory control.

In addition to the above described control and test samples, a positive control reference toxicant test using copper chloride (375, 750, 1500, 3000, and 6000 μg/L) was conducted concurrently to estimate the sensitivity of the test organisms in relation to those historically tested at the laboratory.

#### *Hyalella azteca*

*Hyalella azteca* were obtained from Aquatic Indicators (St. Augustine, FL); the amphipods were 8 days old upon arrival. The *Hyalella* were acclimated to test conditions prior to test initiation in order to promote and confirm animal health. During the acclimation phase, the amphipods were observed for mortality and any indicators of stress, such as abnormal swimming behavior or discoloration. Mortality was considered significant if it was greater than 10 % during the holding and acclimation periods.

*Hyalella* were exposed to reference sediment (mixed or unmixed) or sediment spiked with 10,000 mg/kg TRWP for 42 days, in accordance with methods recommended by the U.S. Environmental Protection Agency (USEPA) (U.S. EPA 2002). The exposure chamber and conditions were maintained and monitored as described for treatment of *Chironomus*, with 12 replicates per treatment group. Four of the 12 replicates were used for intermediate endpoint evaluation (day 28) including survival and growth. On day 28, the amphipods from the remaining eight test chambers were placed into clean jars without sediment; a nitex screen was added as a clean substrate. On day 35, each test chamber was evaluated for reproduction; the number of young produced in each chamber was recorded and the young removed. At day 42, the additional number of young produced was recorded for each test chamber, as well as the number of adult amphipods and their gender. Growth was determined by dry weight after oven-drying of the amphipods.

In addition to the above described control and test samples, a positive control reference toxicant test using copper chloride (100, 200, 400, 800, and 1600 μg/L) was conducted concurrently to estimate the sensitivity of the test organisms in relation to those historically tested at the laboratory.

### Elutriate exposures

#### *Ceriodaphnia dubia*

The *Ceriodaphnia dubia* used for this study were obtained from internal laboratory culture. Prior to test initiation, neonatal water fleas (<24 h old) were isolated from brood stock cultures and placed in individual holding cups containing clean culture water and food. Cultures were maintained in a temperature controlled room at 25 ± 1 °C. Isolated females were transferred daily to new cups containing fresh water and food. Neonates produced within the 24 h prior to testing were selected for chronic toxicity if produced by individuals that had at least three broods averaging eight or more neonates each.

*Ceriodaphnia* were tested for chronic toxicity over a period of 7 days following methods recommended by the USEPA (U.S. EPA 2002). The organisms were treated using sediment elutriate, prepared as described above, from 10,000 mg/kg spiked sediment. Measurements of pH, dissolved oxygen, temperature and conductivity were measured and recorded for each treatment and control. The test consisted of ten replicates exposed to either TRWP sediment elutriate, control sediment elutriate, or control laboratory water (moderately hard water). Test solutions were renewed and organisms fed once daily. Water quality was monitored daily in both freshly prepared test renewal solution and test solution collected from the test chambers. Survival status and reproductive output were recorded once per day. At test termination (7 days), final observations were made and organisms discarded.

In addition to the above described control and test samples, a positive control reference toxicant test using copper chloride (12.5, 25, 50, 100, 200 μg/L) was conducted concurrently to estimate the sensitivity of the test organisms in relation to those historically tested at the laboratory.

#### *Pimephales promelas*

Fathead minnow embryos and larval fish were purchased from Aquatic Biosystems (Fort Collins, CO). Upon receipt, each batch of animals was acclimated to the proper test temperature of 25 ± 1 °C prior to test initiation. Water quality, including conductivity, temperature, dissolved oxygen, and pH, in the larval fish and embryo holding chambers was monitored daily during holding.

To evaluate chronic toxicity in fathead minnows, the hatching success, survival and growth (mass and length) of fathead minnows exposed to the test material (TRWP sediment elutriate) were assessed over a period of 32 days, according to OECD TGD 210 methods and criteria (OECD 1992). The test was initiated with unhatched embryos ranging from 52 to 56 h post-fertilization. Controls included a reference sediment elutriate and a laboratory water (moderately hard water) control. Testing was conducted using six replicate test chambers containing 200 mL of test solution maintained at 25 ± 1 °C with a 16:8 h light:dark cycle. Each test chamber contained ten fish embryos of healthy appearance at test initiation. All test chambers were initiated with continuous, light aeration to maintain dissolved oxygen content in an acceptable range. Water renewals were performed every 8 days due to limited test material.

To assess toxicity, hatching status and survival of post-hatch larvae was recorded daily. At test termination, final observations were made and test animals were prepared for weight and length determination. Fork length of each fish, measured from the tip of the snout to the posterior end of the middle caudal fin rays to the nearest 0.5 mm, was recorded at test termination. Fish growth was determined by measuring the pooled dry weight of all fish in each test chamber.

In addition to the above described control and test samples, a positive control reference toxicant test using copper chloride (15, 30, 60, 120, and 240 μg/L) was conducted concurrently to estimate the sensitivity of the test organisms in relation to those historically tested at the laboratory.

### Statistical analyses

#### Sediment and elutriate test comparisons

Survival (proportional) data were arcsin square-root transformed prior to analysis. Growth data was log-transformed as needed to satisfy statistical assumptions. Reproduction data was not transformed for any species prior to analysis. Prior to and following transformation, all datasets were examined for normality using either the Shapiro–Wilk, or the D’Agostino & Pearson omnibus normality test. Homogeneity of variance was determined using either the Bartlett’s test or F-ratio test.

*Ceriodaphnia dubia*, *Chironomus dilutus, Hyalella azteca,* and *Pimephales promelas* test data were analyzed with GraphPad Prism^®^ Statistical Software, Version 4.02. Statistical comparisons to determine whether the TRWP impacted *Chironomus dilutus* and *Hyalella azteca* performance were conducted between the spiked sediment and mixed reference sediment. Differences between other treatments were evaluated for comparative purposes only. Likewise, statistical comparisons to determine the effects of TRWP on fathead minnows were conducted between the elutriate control and the TRWP-spiked elutriate.

A one-way analysis of variance (ANOVA) was performed to determine if there were any significant differences in treatments means for the toxicity tests that were performed (Neter et al. 1990). If the ANOVA indicated that there was at least one significant difference at 95 % confidence level (*p* < 0.05), the Tukey pairwise multiple comparison tests were performed to identify the specific significant differences. The assumption of a normal distribution was verified for each ANOVA using probability plots of the data for each treatment group.

#### Reference toxicant tests

Statistical analyses for standard reference toxicant tests were conducted using CETIS Version 1.6.3revE. Normality of the data was established by Shapiro–Wilk’s Normality Test and equality of variance by either Levene’s or Bartlett’s test. A medial lethal concentration (LC50) value was then calculated using either the Trimmed Spearman-Karber or Linear Interpolation methods. Statistical methods were chosen based on methodology for data analysis as outlined by the USEPA (U.S. EPA 2002).

## Results

### Whole sediment exposures

#### *Chironomus dilutus*

A summary of *Chironomus* results is presented in Table 1. On day ten, survival of chironomids did not differ significantly among any treatments (*p* = 0.141); mean survival in laboratory control sediment was 67 %, compared to mixed reference sediment and TRWP-spiked sediment which had mean survival percentages of 63 and 57 %, respectively. Survival was greater in unmixed reference sediment (90 %). *Chironomus growth*, as indicated by AFDW, in the TRWP-spiked sediment did not differ significantly from the mixed reference sediment. *Chironomus* emergence also did not differ among treatment groups (*p* = 0.350), based on an emergence of 7.4 adults in laboratory control sediment compared to 6.6, 6.6 and 4.8 in mixed reference sediment, unmixed reference sediment and TRWP-spiked sediment groups, respectively. The reference toxicant test demonstrated that the *Chironomus* were sufficiently sensitive based on a LC50 of 54.1 μg/L (95 % CI = 0.00114–191 μg/L) which was within the historical mean values for the laboratory.

**Table 1:** *Chironomus dilutus*—mean survival, weight and emergence

Treatment	Day 10 mean % survival (SD)	Day 10 mean AFDW per organism (mg) (SD)	Mean # of adults emerged: days 20–35 (SD)
Laboratory control	67 (27)	0.231 (0.032)	7.4 (2.5)
Reference sediment (unmixed)	90 (7)	0.323 (0.103)	6.6 (1.5)
Reference sediment (mixed)	63 (31)	0.254 (0.054)	6.6 (2.7)
TRWP spiked sediment	57 (11)	0.199 (0.029)*	4.8 (2.2)

*AFDW* ash free dry weight

* Statistically significant decrease relative to the unmixed reference sediment (Tukey pairwise multiple comparison test, *p* = 0.028)

The laboratory control mean survival of 67 % was slightly less than the OECD and US EPA criteria of 70 % and the laboratory control emergence of 62 % was slightly less than the OECD criteria of 70 % but greater than the US EPA criteria of 50 %. Control results were deemed acceptable for reporting because sufficient statistical power was achieved to identify significant differences and all water quality parameters were within proper ranges as defined in the test protocol.

#### *Hyalella azteca*

A summary of *Hyalella* results is presented in Table 2. For all endpoints of toxicity at both time points (day 28 and day 42), there was no statistically significant difference between any of the treatment groups, indicating that TRWP-spiked sediment did not adversely affect *Hyalella azteca* survival, growth or reproduction. The reference toxicant test demonstrated that the *Hyalella* were sufficiently sensitive based on a LC50 of 400 μg/L (95 % CI = 336–476 μg/L) which was within the historical mean values for the laboratory.

**Table 2:** *Hyalella azteca*—mean survival, weight, and reproduction

Treatment	Day 28 mean % survival (SD)	Day 28 mean dry weight per organism (mg) (SD)	Day 42 mean % survival (SD)	Day 42 mean dry weight per organism (mg) (SD)	Day 42 mean # young per female (SD)
Laboratory control	95 (12)	0.354 (0.021)	86 (20)	0.456 (0.069)	6.6 (2.8)
Reference sediment (unmixed)	96 (12)	0.366 (0.068)	89 (10)	0.422 (0.081)	2.8 (1.8)*
Reference sediment (mixed)	97 (6)	0.276 (0.007)	86 (16)	0.404 (0.031)	4.1 (0.8)*
TRWP spiked sediment	96 (5)	0.298 (0.050)	90 (12)	0.409 (0.063)	3.0 (1.4)*

* Statistically significant decrease relative to lab control based on Tukey pairwise multiple comparison test (unmixed, *p* = 0.002; mixed, *p* = 0.049; TWP, *p* = 0.003)

### Sediment elutriate exposures

#### *Ceriodaphnia dubia*

A summary of *Ceriodaphnia* results is presented in Table 3. For all endpoints of toxicity, including survival and reproduction at day 7, there was no statistically significant difference between the lab control and the TRWP-spiked elutriate. This indicates that TRWP did not adversely affect *Ceriodaphnia dubia* following chronic exposure. However, there was a significant difference between the elutriate control and the laboratory control. The reference toxicant test demonstrated that the *Ceriodaphnia* were sufficiently sensitive based on a LC50 of 44.4 μg/L (95 % CI = 33.8–58.3 μg/L) and an EC50 (reproduction) of 40.9 μg/L (95 % CI = 33.1–54.1 μg/L), both of which were within the historical mean values for the laboratory.

**Table 3:** *Ceriodaphnia dubia*—mean 7-day survival and reproduction

Treatment	Mean % survival (SD)	Mean # neonates (SD)
Laboratory control	100 (0)	28 (2.9)
Elutriate control	100 (0)	7.3 (4.4)*
TRWP spike elutriate	100 (0)	26 (2.3)

* Method control (elutriate control) reproduction was significantly less than the laboratory control (*p* < 0.0001) and the TWP treatment (*p* < 0.0001) based on Tukey pairwise multiple comparison test

#### *Pimephales promales*

A summary of *Pimephales* results is presented in Table 4. There were no statistically significant differences between hatch success or growth parameters among the treatment groups and no significant difference in survival between the TRWP-spiked sediment and the elutriate control. The reference toxicant test demonstrated that the *Pimephales* were sufficiently sensitive based on a LC50 of 126 μg/L (95 % CI = 108–146 μg/L) and an EC50 (dry biomass) of 108 μg/L (95 % CI = 75–160 μg/L), both of which were within the historical mean values for the laboratory.

**Table 4:** *Pimephales promelas*—hatch-success, survival and growth

Treatment	Day 32 mean % hatch success (SD)	Mean % survival^a^ (SD)	Mean biomass (mg/organism)^b^ (SD)	Mean length (mm/organism)^c^ (SD)
Laboratory control	92 (8)	76 (9)	1.75 (0.21)	11 (0.53)
Elutriate control	87 (18)	69 (16)	2.06 (0.61)	12 (0.91)
TRWP spiked elutriate	95 (8)	55 (17)*	1.9 (0.22)	13 (2.03)

* Statistically significant decrease relative to the Lab control (Tukey pairwise multiple comparison test, *p* = 0.016)

^a^Mean survival calculation based on post-hatch survival

^b^Mean biomass calculation based on initial number of hatched fishes

^c^Mean length calculation based on 32-day data

## Discussion

Previous studies with TRWP have indicated that acute exposures to up to 10,000 mg/kg in sediment do not result in toxicity in algae, daphnids, or fathead minnows (Marwood et al. 2011). However, it has been suggested that chronic exposure to TRWP in the environment could cause toxicity, either as a result of increased duration of exposure to low levels of leached chemicals, or leaching of chemicals after longer incubation periods. Studies employing methods that promote leaching of chemicals from TRWP (e.g. heating of the water/TRWP mixture, use of organic extracts) have indicated some potential for toxicity under acute exposure scenarios (Marwood et al. 2011; Wik and Dave 2006; Gualtieri et al. 2005b). However, this approach may result in bolus leaching, and thus higher exposure concentrations for individual chemicals, rather than the gradual leaching that would occur under environmental conditions of chronic exposure. The purpose of this study was to determine if chronic exposure of both sediment and water-dwelling aquatic species to TRWP in sediment could result in deleterious effects on aquatic organism survival, growth or reproduction.

Of the sediment-dwelling species evaluated in this study, TRWP did not cause toxicity in any species tested. In *Chironomus* exposed to TRWP-spiked sediment, there was an approximately 20 % reduction in growth, as measured by AFDW, when compared to mixed reference sediment. However, this reduction was not statistically significant and there was no effect on reproduction or survival. There was no effect on any endpoint of toxicity in *Hyalella azteca* exposed to TRWP-spiked sediment. The results in *Hyalella* are consistent with Wik et al. (2008), wherein the researchers evaluated the toxicity of sediment and site water from a variety of locations to *Hyalella azteca* and observed no toxicity even in areas determined to have high concentrations of tire wear debris based on the detection of organic zinc as a chemical marker (Wik et al. 2008). To date, no other studies have been performed to assess the toxicity of any tire particles (either tread particles or TRWP) to sediment-dwelling species, despite sediment being the primary reservoir for TRWP in the natural environment.

Of the water-dwelling species evaluated in this study, TRWP did not cause toxicity in either species tested. Previous studies have indicated that *Ceriodaphnia dubia* are particularly sensitive to leachates from tires (Wik and Dave 2009; Wik et al. 2009). Wik et al. (2009) conducted sequential leaching of tread particles and treated a variety of aquatic species with the leachates. These researchers found that *Ceriodaphnia dubia* were the most sensitive of the species tested to the effects of the leachates. However, the leachates were sequentially less toxic, indicating that with water renewal, as would occur in the natural environment, the toxicity of the tread particles decreased (Wik et al. 2009). Furthermore, Wik et al. utilized only an aqueous extract of tread particles. TRWP have been determined to be significantly different than naïve tread particles in chemical composition, and therefore TRWP may release different chemicals into the aqueous solution than those studies that use only tread particles (Kreider et al. 2010). In addition, the use of an aqueous extract in the absence of sediment, which represents the main reservoir for TRWP in the natural environment and which may limit the bioavailability of chemicals to the aquatic species, may further explain the differences between the results found here and those reported by Wik et al. Marwood et al. demonstrated that the presence of sediment can attenuate the toxic response of heated TRWP elutriate (Marwood et al. 2011). Though not statistically significant, there was an approximately 20 % reduction in survival in *Pimephales* exposed to TRWP-elutriate when compared to elutriate control. To date, there have been no other studies performed on TRWP or tread particles in *Pimephales promelas*. Studies on whole tires submerged in water bodies have found that leaching from tire rubber was typically not lethal following acute exposures to *Pimephales* (Abernathy 1994; Nelson et al. 1994).

It appeared that the method of sediment TRWP spiking via homogenization may have reduced performance in several of the endpoints examined in both whole sediment and elutriate tests. Specifically, *Ceriodaphnia* reproduction, *Hyalella* 28- and 42-day dry weight and *Hyalella* reproduction in the mixed unspiked reference sediment and elutriate method controls were all found to be significantly reduced compared to the laboratory control. It is hypothesized that the breakdown and release of fine clay particles within the reference sediment as was visually apparent based on excessive turbidity in the elutriate and texture of the whole sediment may have had a negative impact on these tests. Additionally, it is possible that some of the metals in the control sediment were solubilized into the elutriate during the sediment mixing activity. In future study designs it may be advisable to conduct a pre-test metals analysis of the unmixed and mixed elutriates to understand if metals mobilization could be a confounding factor.

Overall, the toxicity observed from chronic exposure to TRWP-containing sediment or sediment elutriate was low. In comparison to existing data from studies designed to maximize or promote leaching of chemicals from the rubber matrix, the observed effects in this study were minimal. Those species (e.g. *Ceriodaphnia dubia*) previously identified as sensitive to leachates from tread particles were unaffected under environmentally relevant exposure conditions provided in this study. In other studies, the use of heated water or organic extracts has resulted in LC50 s ranging from approximately 0.5 to >10 g/L following only acute exposure (Gualtieri et al. 2005b; Wik and Dave 2006). However, the bolus release resulting from the studies’ designs do not mimic a longer term release of chemicals and therefore are not representative of chronic exposure conditions.

Given the high concentration of TRWP tested in this study and the non-significant, minor effects on survival and reproduction of various aquatic species under chronic exposure conditions, biologically relevant impacts from TRWP in populations of aquatic plants or animals in environments are unlikely. Small changes in measured endpoints, such as growth, lethality, or reproduction, under laboratory controlled scenarios may not necessarily result in an impact on the survival of that same population or species in an ecosystem (Suter 1993; Suter et al. 2000). When extrapolating effects from laboratory exposures to the natural environment, there is low confidence that long-term deleterious effects would occur in the environment when measured effects are small. In this study, no statistically significant effect on any endpoint was greater than 20 %, which is considered *de minimis* by some ecologists and an indication that TRWP is unlikely to have an impact on aquatic biota in the environment (Suter et al. 2000).

This study currently represents the best available information for understanding the potential for chronic toxicity of TRWP; the study design utilizes the best representation of what occurs in the environment including both the incorporation of sediment for exposure to water-dwelling organisms and the use of TRWP instead of tread particles. Nevertheless, a few limitations have been identified and considered. First, logistical constraints prevented the testing of additional aquatic species. However, in other studies of tire leachates, the *Ceriodaphnia dubia* was found to be equal to or more sensitive than *Pseudokirchneriella subcapitata*, *Daphnia magna*, *Xenopus laevis*, and *Danio rerio* when studied using the same experimental design (Wik et al. 2009; Gualtieri et al. 2005b; Mantecca et al. 2007). Although these comparisons were made under conditions of short term exposure, it is anticipated that relative sensitivity to chronic exposure will be similar for these species and therefore, these other species are also unlikely to be affected by chronic exposure to TRWP. Aquatic toxicity testing on leaching of rubber hoses and submerged whole tires or tire shreds showed toxicity to *Oncorhynchus mykiss*, but not *Pimephales*, indicating that *Pimephales* may not be the fish species most sensitive to rubber chemicals (Abernathy 1994; Stephensen et al. 2005; Day et al. 1993; Abernathy et al. 1996; Goudey and Barton 1992). However, the results from these studies may not be representative of toxicity that occurs with TRWP, as whole tires and general rubber goods, such as rubber hoses, contain different/additional chemical constituents than tread rubber.

The second limitation in this study relates to the duration of incubation of the TRWP in the sediment–water mixture during the generation of the sediment elutriate. In this study the water was equilibrated with the TRWP for 24 h before the sediment elutriate was removed and used for treatment of the water-dwelling species. In the environment, it is anticipated that the TRWP will be washed into receiving water bodies through stormwater discharge and deposited, primarily, into sediment. For receiving waters where the water is renewed frequently, one could anticipate that the initial leaching of chemical would result in the greatest toxicity and that as chemicals are removed from the rubber matrix and subsequently diluted through water renewals, the toxicity of the TRWP would decrease. In fact, Wik et al. demonstrated that toxicity of tread particles is reduced upon sequential leaching of the same material (Wik et al. 2009). Therefore, this study design, while somewhat limited in the duration of incubation of TRWP in water, should characterize the potential for toxicity from initial leaching of chemicals from the rubber matrix. However, there is the potential for stagnant water bodies to receive TRWP; this scenario, while likely uncommon, is not represented by this study design.

## Conclusions

Based on the results presented here and in spite of the minor limitations on study design, TRWP present in road dust is of minor importance with respect to aquatic toxicity. Although small decrements in growth and survival were found in *Chironomous* and *Pimephales*, respectively following chronic exposure to 10,000 mg/kg TRWP in sediment, these effects were not significant and are unlikely to have deleterious consequences on populations of these species in natural ecosystems.
