# Correction to: Chronic toxicity of tire and road wear particles to water- and sediment-dwelling organisms

**DOI:** 10.1007/s10646-020-02227-y

**Published:** 2020-05-16

**Authors:** Julie M. Panko, Marisa L. Kreider, Britt L. McAtee, Christopher Marwood

**Affiliations:** 1 ChemRisk, 20 Stanwix Stree Stuite 505, Pittsburgh, PA 15222 USA; 2 NovaTox Inc., 10 Crane Avenue, Guelph, ON N1G 2R2 Canada

Correction to: Ecotoxicology (2013) 22:13–21

10.1007/s10646-012-0998-9

The article Chronic toxicity of tire and road wear particles to water- and sediment-dwelling organisms, written by Julie M. Panko, Marisa L. Kreider, Britt L. McAtee, and Christopher Marwood, was originally published electronically on the publisher’s internet portal on 22 September 2012 without open access. With the author(s)’ decision to opt for Open Choice the copyright of the article changed on May 2020 to © The Author(s) 2020 and the article is forthwith distributed under a Creative Commons Attribution 4.0 International License (https://creativecommons.org/licenses/by/4.0/), which permits use, sharing, adaptation, distribution and reproduction in any medium or format, as long as you give appropriate credit to the original author(s) and the source, provide a link to the Creative Commons licence, and indicate if changes were made.

**Publisher’s Note** Springer Nature remains neutral with regard to jurisdictional claims in published maps and institutional affiliations.

